# Deformation-Assisted Joining of Sheets to Tubes by Annular Sheet Squeezing

**DOI:** 10.3390/ma12233909

**Published:** 2019-11-26

**Authors:** Luis M. Alves, Rafael M. Afonso, Frederico L.R. Silva, Paulo A.F. Martins

**Affiliations:** Instituto de Engenharia Mecânica, Instituto Superior Técnico, Universidade de Lisboa, Av. Rovisco Pais, 1049-001 Lisboa, Portugal; luisalves@tecnico.ulisboa.pt (L.M.A.); rafael.afonso@tecnico.ulisboa.pt (R.M.A.);

**Keywords:** joining, forming, sheet–tube connections, experimentation, modelling and simulation

## Abstract

This paper is built upon the deformation-assisted joining of sheets to tubes, away from the tube ends, by means of a new process developed by the authors. The process is based on mechanical joining by means of form-fit joints that are obtained by annular squeezing (compression) of the sheet surfaces adjacent to the tubes. The concept is different from the fixing of sheets to tubes by applying direct loading on the tubes, as is currently done in existing deformation-assisted joining solutions. The process is carried out at room temperature and its development is a contribution towards ecological and sustainable manufacturing practices due to savings in material and energy consumption and to easier end-of-life disassembly and recycling when compared to alternative processes based on fastening, riveting, welding and adhesive bonding. The paper is focused on the main process parameters and special emphasis is put on sheet thickness, squeezing depth, and cross-section recess length of the punches. The presentation is supported by experimentation and finite element modelling, and results show that appropriate process parameters should ensure a compromise between the geometry of the mechanical interlocking and the pull-out strength of the new sheet–tube connections.

## 1. Introduction

In recent years, there has been a growing utilization of deformation-assisted joining processes driven by an increasing demand of assembling lightweight components. Deformation-assisted joining processes are classified into three distinct groups according to their operating principles:

(i) Based on mechanical, hydraulic, and magnetic loading, such as clinching [1,2], self-pierce riveting [3], sheet-bulk compression [4], hydraulic forming [5], and electro-magnetic forming [6];
(ii) Based on solid-state welding, such as friction stir welding [7], friction spot welding [8] and explosive welding [9];
(iii) Based on fusion welding combined with plastic deformation, such as resistance spot and projection welding [10] and weldbonding [11].

Some of the above-mentioned processes have been extensively investigated for joining sheets and tubes made from similar or dissimilar materials. The state-of-the-art reviews by Mori et al. [12] and Groche et al. [13] provide detailed information on the most significant developments and applications of deformation-assisted joining processes to connect sheets and tubes.

**Figure 1:**
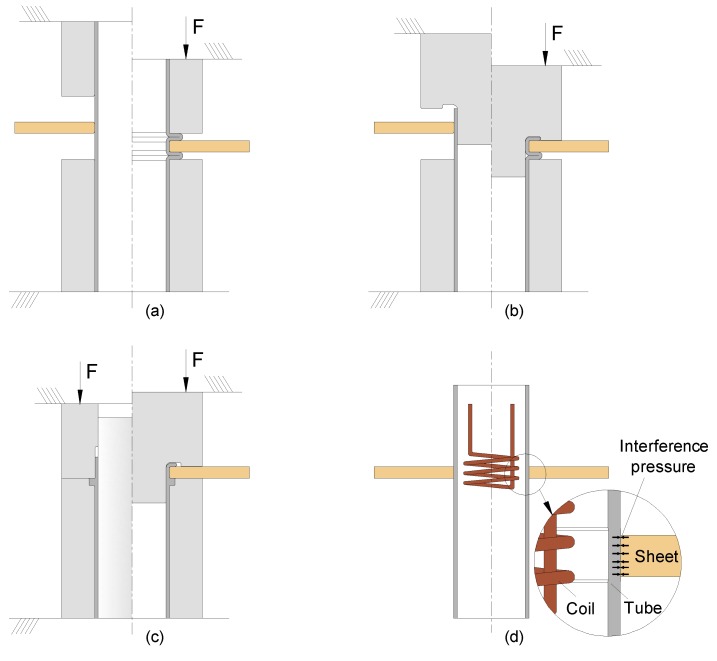
Deformation-assisted joining of sheets to tubes by mechanical joining. (**a**) Form-fit joints produced by tube compression beads formed by local buckling. (**b**) Form-fit joints produced by a combination of tube compression beads and flaring. (**c**) Form-fit joints produced by a combination of partial sheet-bulk compression of tubes and flaring. (**d**) Force-fit joints produced by the pressure that remains after elastic recovery from magnetic loading.

Despite the progresses in sheet-to-sheet and tube-to-tube connections, the joining of sheets-to-tubes is still preferentially accomplished by means of conventional fastening and welding technologies. Little progress has been made in the application of deformation-assisted joining to sheet–tube connections, aside from recent developments involving the utilization of form-fit mechanical joints based on compression beads formed by local buckling [14], on the combination of tube compression beads with flaring [15], and on the combination of partial sheet-bulk compression of tubes with upsetting [16] or flaring [17] (Figure 1a–c).

The utilization of force-fit mechanical joints that exclusively rely on the pressure that remains on the contact interfaces after elastic recovery from mechanical [18], hydraulic [5], or magnetic loading [6] is not a feasible solution for the fixing of sheets to tubes due to the limited contact surfaces provided by the small thickness sheets that are commonly used in the industry (Figure 1d). Similar constraints apply to new developments for the joining of thick blocks (or plates) to tubes by compressing the upper end of a connecting block flange [19,20] because of the need to produce the connecting flange by machining.

Regarding the connection of sheets to tubes, which is the main objective of this paper, it is worth mentioning that all the previously mentioned deformation-assisted joining processes based on mechanical form-fit joints [14,15,16,17] involve multiple operations. This undercuts their advantages in material consumption, energy requirements, and end-of-life recycling with disadvantages related to efficiency and costs that are better ensured by conventional technologies based on fastening, welding, and adhesive bonding. Besides the limitations resulting from existing processes based on mechanical form-fit joints being carried out in multiple operations, there is also the risk of failure by cracking in the compression beads produced by local buckling. This reduces their applicability to tubes with low fracture toughness [21].

**Figure 2:**
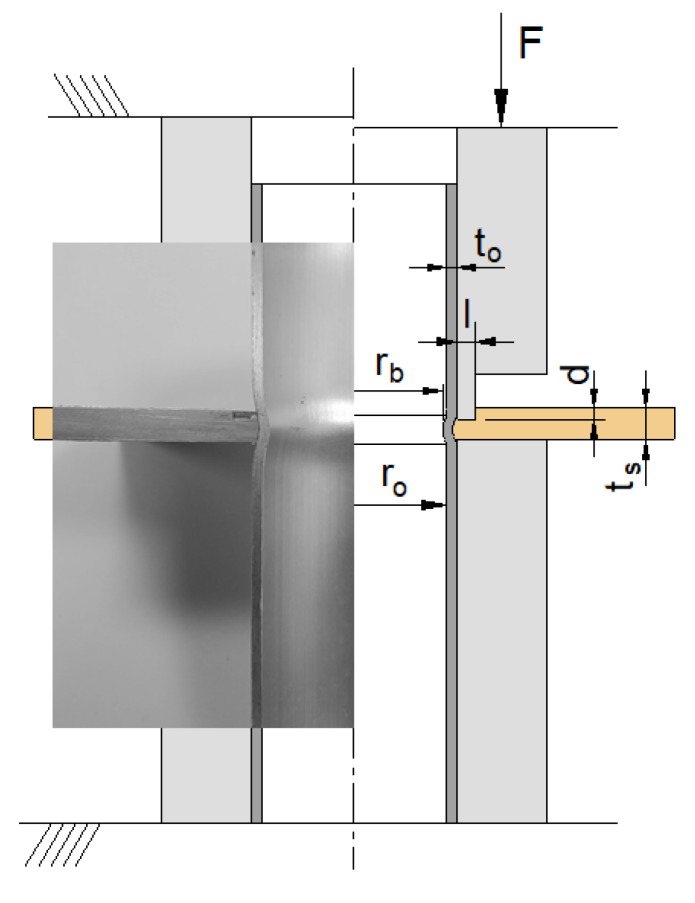
A representation of the deformation-assisted joining of sheets to tubes by annular sheet squeezing at the open and closed positions [22]. A photograph of a longitudinal cross section of a test specimen is enclosed.

All of the above-mentioned problems prompted the authors to develop a new process for fixing sheets to tubes at room temperature that is based on an entirely new mechanical joining concept in which loading is applied on the sheet surface instead of the tube itself [22]. The process is schematically shown in Figure 2, and consists of squeezing (compressing) the annular surface of the sheet adjacent to the tube in order to ensure the material from the sheet flows inwards and is shaped as a form-fit joint with good mechanical interlocking between the sheet and the tube.

The new joining process is performed in a single punch stroke and requires both the sheet and the tube to have some degree of ductility to plastically shape the form-fit joint. The optimum operating conditions require the material of the tube to have a lower elastic modulus than that of the sheet, so that the pressure remains on the contact interface after producing the form-fit joint as a result of the more pronounced elastic recovery of the tube in the direction of the sheet. Otherwise, the resulting form-fit joint may end up slightly loose as in the case of sheet–tube connections made from dissimilar materials (e.g., metals and polymers or composites) with very different elastic modulus. Another requirement of the new proposed joining process is the necessity of the sheet strength being similar or higher to that of the tube to allow for easy shaping of the inner tube bead that is needed to produce the form-fit mechanical joint.

Finally, it is worth mentioning that the new joining process circumvents the previously-mentioned difficulties resulting from the utilization of tubular materials with low fracture toughness.

This paper is focused on the main process parameters of deformation-assisted joining of sheets to tubes by annular sheet squeezing, hereafter designated as ‘mechanical joining of sheets to tubes’. Special emphasis is put on the sheet thickness $t_{s}$, squeezing depth d, and cross-section recess length l of the punches due to their influence on the inner tube bead shape of the form-fit joint and on the quality and performance of the mechanical joint between the sheet and the tube. The presentation includes results from experimentation and finite element modelling, and from destructive pull-out tests that were carried out to determine the maximum force that the new joints can withstand before failing.

## 2. Materials and Methods

### 2.1. Materials and Flow Curves

The work on the mechanical joining of sheets to tubes by annular sheet squeezing was carried out in two different aluminum alloys. The sheets were made of aluminum AA5754-H111 with a 5 mm thickness and the tubes were made of aluminum AA6063-T6 with an outer radius of 16 mm and a 1.5 mm wall thickness.

**Figure 3:**
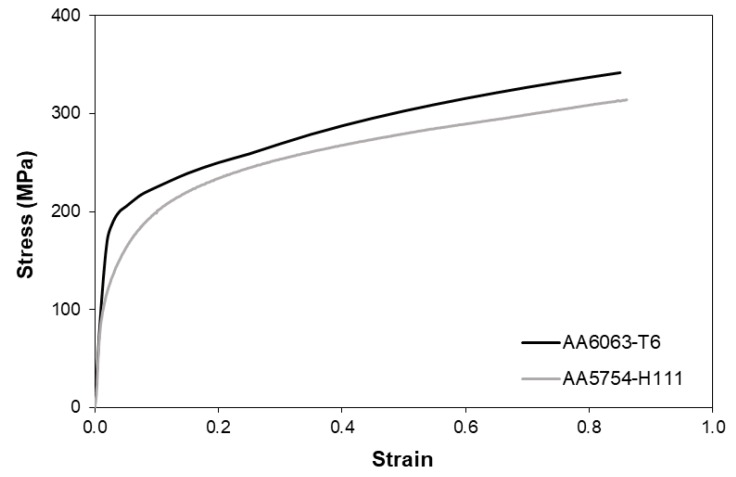
Flow curves of the two aluminum alloys utilized in the experiments.

The flow curves of the two materials were obtained by combining tensile and stack compression tests [23] performed in a hydraulic testing machine with a cross-head speed of 5 mm/min. The tensile tests allowed characterization of the stress response of the materials for small values of strain, whereas the stack compression tests were utilized to determine the stress response for larger values of strain, beyond plastic instability in tension, following a procedure similar to that of Silva et al. [4]. The flow curves of the AA5754-H111 sheets and AA6063-T6 tubes are shown in Figure 3.

The reason for using two materials with similar flow curves and nearly identical elasticity moduli (with differences within the range of 68 to 68.9 GPa) was to allow for the study of the performance of the form-fit joints alone and independently of the interfacial pressure, which develops on the sheet–tube contact surface in case of form-fit joints made from materials with a different elasticity modulus.

### 2.2. Experimental Tests

In their original paper on the mechanical joining of sheets to tubes by annular sheet squeezing [22], the authors put emphasis on the influence of the cross-section recess length l of the punch on plastic material flow inside the sheet thickness. The squeezing depth d was kept constant and a deformation-zone geometry parameter $\Delta =t_{s}/l$, defined as the ratio of the sheet thickness $t_{s}$ to the cross-section recess length l, was introduced to characterize material flow and identify the deformation modes associated with acceptable and unacceptable joints.

In a subsequent paper [24], the authors focused on the complementary work plan by analyzing the influence of the squeezing depth d and keeping the cross-section recess length l of the punch at a fixed value. The investigation allowed the characterization of the physics behind material separation at the cross-section recess corner of the punch and to reach a better understanding of the influence of the squeezing depth d on the pull-out destructive strength of the joints.

The work plan giving support to this paper takes the combined influence of d and l into account. The goal is to understand how changes in both variables at the same time will influence material flow and the overall pull-out performance of the joints so that a procedure can be reached to determine the combination of squeezing depth d and cross-section recess length l that is capable of ensuring the best form-fit joint for a supplied set of geometries and materials.

Such a procedure has not been addressed in previous papers and is of paramount importance when producing form-fit joints in dissimilar materials in which the strength of the tube is similar or slightly higher than that of the sheet. 

The overall methodology utilized in the experimental tests consisted on the following steps:

1. The tube and the sheet were first cut to the required sizes;
2. A hole with a diameter equal to the outer tube diameter was drilled in the sheet;
3. The tube and the sheet were then placed in the tooling system installed in a hydraulic testing machine INSTRON SATEC 1200 kN;
4. The hydraulic testing machine moved the punch downwards in order to locally compress the sheet up to a total squeezing depth
5. Steps 1 to 4 were repeated several times in order to vary the sheet thickness *t_s_*, the cross-section recess length of the punch, and the squeezing depth *d* according to the values listed in Table 1. All the tests were performed at room temperature;
6. After finishing the tests, selected samples were cut along their axial cross-sectional planes in order to analyze their mechanical form-fit joints, to take photographs, and to perform measurements;
7. Finally, another set of selected samples was subjected to destructive pull-out tests in order to evaluate the overall performance of the joints.

Table 1 presents a summary of the experiments on mechanical joining together with the major geometrical specifications of the sheets and tubes that were utilized in the investigation. The influence of the sheet thickness $t_{s}$ was taken into consideration by extending the experimental work to sheets with a smaller thickness ($t_{s}=2.5$ mm) than the nominal supplied thickness $t_{s}=5$ mm.

**Figure 4:**
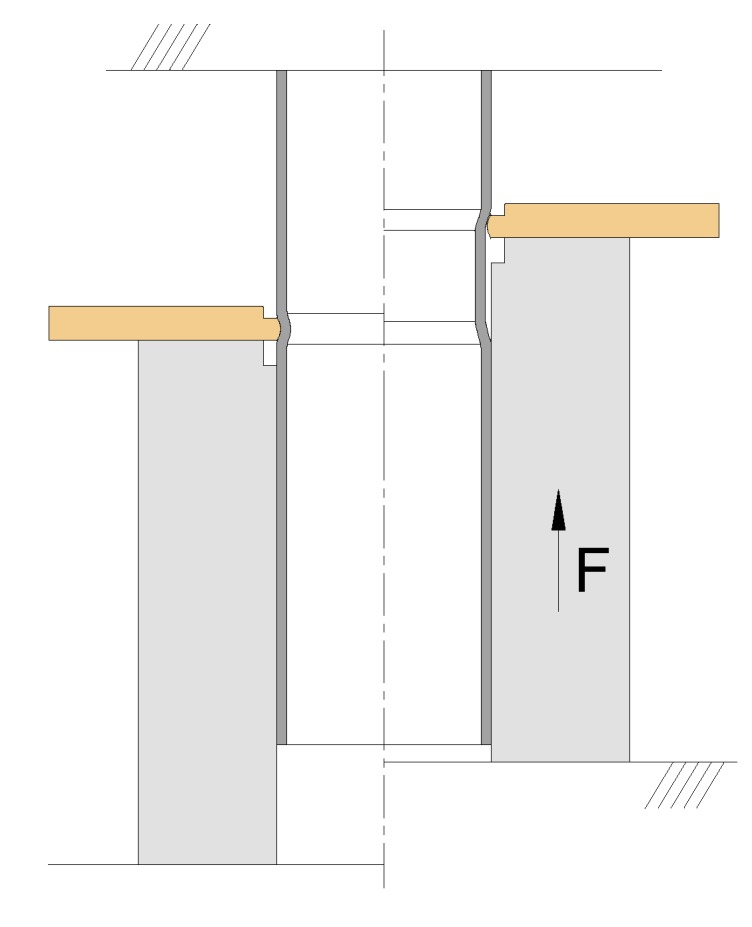
A representation of the experimental pull-out destructive setup before (left) and during testing (right).

Figure 4 presents a schematic representation of the experimental setup that was utilized to evaluate the performance of the new form-fit joints. The joints were subjected to destructive pull-out tests in which the sheet was detached from the tube, and the objective was the determination of the maximum force *F* that the joints can withstand before failing.

### 2.3. Finite Element Modelling

The in-house finite element computer program I-form was utilized to analyze the mechanical joining of sheets to tubes by annular sheet squeezing. The computer program is based on the finite element flow formulation [25], and its implementation follows the extension of the rigid-plastic Markov’s principle [26] of minimum plastic work to include incompressibility and contact between deformable bodies,

(1) $$\Pi =\int_{V}\bar{\sigma }\;\dot{\bar{\varepsilon }}\;dV+\frac{1}{2}K\;\int_{V}{\dot{\varepsilon }}_{v}^{2}\;dV-\int_{S_{T}}T_{i}u_{i}\;dS+\int_{S_{f}}\left(\int_{0}^{\left|u_{r}\right|}\tau_{f}du_{r}\right)\;dS+\frac{1}{2}K_{1}\sum_{c=1}^{N_{c}}{\left(g_{n}^{c}\right)}^{2}+\frac{1}{2}K_{2}\sum_{c=1}^{N_{c}}{\left(g_{t}^{c}\right)}^{2}$$

In the first term of Functional (1), the symbols $\bar{\sigma }$ and $\dot{\bar{\varepsilon }}$ denote the effective stress and the effective strain rate, respectively,

(2) $$\bar{\sigma }=\sqrt{\frac{3}{2}\sigma_{ij}^{’}\sigma_{ij}^{’}}\;\dot{\bar{\varepsilon }}=\sqrt{\frac{2}{3}{\dot{\varepsilon }}_{ij}{\dot{\varepsilon }}_{ij}}$$

where $\sigma_{ij}^{’}$ is the deviatoric stress tensor and ${\dot{\varepsilon }}_{ij}$ is the strain rate tensor.

In the second term, the symbol ${\dot{\varepsilon }}_{v}$ is the volumetric strain rate, given by

(3) $${\dot{\varepsilon }}_{v}=\delta_{ij}{\dot{\varepsilon }}_{ij}$$

where $\delta_{ij}$ is the Kronecker delta and K is a large positive number utilized to impose incompressibility in volume V by means of a penalty factor. 

The third term of Functional (1) makes use of the surface tractions $T_{i}$ and velocities $u_{i}$ on the surface $S_{T}$, whereas the fourth term takes care of the frictional effects along the contact interface $S_{f}$ between the sheet and tube with the tools. In this work, tools were assumed as rigid bodies, and $\tau_{f}$ and $u_{r}$ denote the friction shear stress and relative sliding velocity of the sheet and tube. The friction shear stress was modelled according to the law of constant friction,

(4) $$\tau_{f}=m\;k$$

where m is the friction factor between the sheets and tubes, taken as 0.1 after checking the predicted forces that best matched the experimental results. The symbol k denotes the flow shear stress.

The fifth and sixth terms account for the contact between the sheet and tube modelled as deformable bodies along their contact interfaces defined by means of $N_{c}$ pairs extracted from the faces of the elements that were utilized in their discretization. The symbols $g_{n}^{c}$ and $g_{t}^{c}$ denote the normal and tangential gap velocities in the contact pairs, which are penalized by large numbers $K_{1}$ and $K_{2}$ to avoid penetration. A more detailed look into the numerical implementation of Functional (1) in the finite element computer program I-form can be found in reference [27].

**Figure 5:**
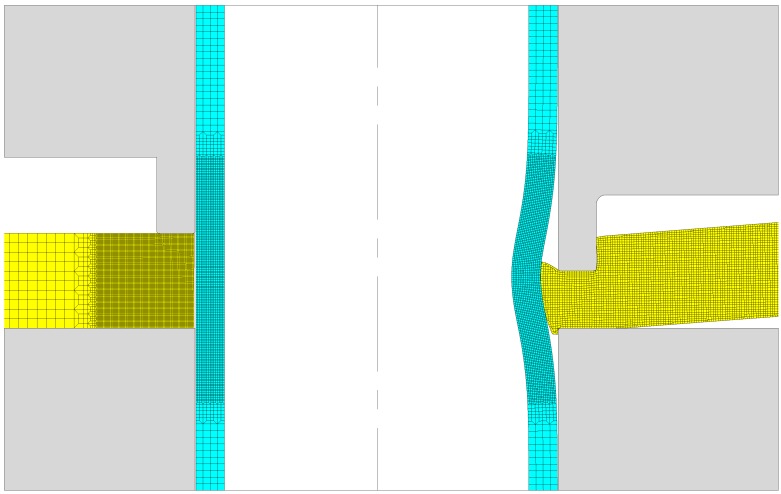
Element meshes before and after fixing the sheet to the tube using mechanical joining by annular sheet squeezing (l=2 mm and d=2 mm and $t_{s}=5$ mm).

Figure 5 shows in detail the finite element model before and after the sheet is mechanically joined to the tube. The finite element models utilized in the numerical modelling of the process made use of rotational symmetry conditions and required discretization of the longitudinal cross-section of the sheets and tubes by means of approximately 20,000 and 800 quadrilateral elements, respectively. The sheet and the tube were modelled as deformable bodies, whereas the tools were modelled as rigid objects and discretized by means of linear contact-friction elements. Several remeshings were carried out to avoid excessive element distortion during annular sheet squeezing.

## 3. Results

**Figure 6:**
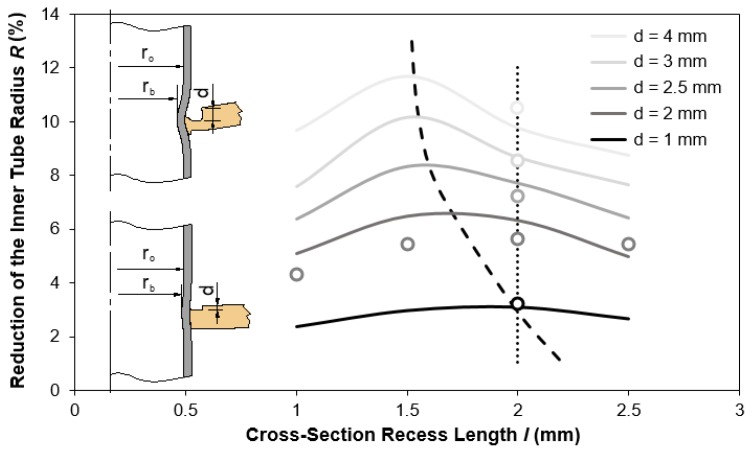
Reduction of the inner tube radius R as a function of the cross-section recess length l of the punch for different values of the squeezing depth d.

Figure 6 shows the finite element computed reduction of the inner tube radius $R=\left(r_{0}-r_{b}\right)/r_{0}$ as a function of the cross-section recess length l of the punch for four different values of the squeezing depth d. Experimental measurements of R for a squeezing depth d=2 mm and a cross recess length l=2 mm are included to assess the validity and reliability of the finite element estimates.

The first conclusion derived from Figure 6 is that small values of d lead to small amounts of material being displaced against the tube, and therefore, to the development of less pronounced inner tube bead geometries of the form-fit joints. In contrast, large values of d give rise to large amounts of material being squeezed against the tube and to more pronounced inner tube bead geometries of the form-fit joints. This is graphically shown in the two schemes that are included in Figure 6.

As seen in Figure 6, a typical R(l) evolution passes through a peak corresponding to the cross-section recess length l that provides the maximum reduction $R_{\max}$ of the inner tube radius for a given squeezing depth d. The dashed curve passing through all these peaks defines the correlation between l and d that maximizes the inner tube bead geometry of the form-fit joints.

To the left of the dashed curve, there is a decrease in the amount of sheet material being squeezed as the cross-section recess length l diminishes. This leads to smaller values of R and to the development of less pronounced inner tube bead geometries of the form-fit joints. At the limit, there will be no form-fit joint. 

Contrary to what one would expect, to the right of the dashed curve, the R(l) evolutions should increase were it not for the squeezed sheet material starting to moving outwards instead of inwards. 

**Figure 7:**
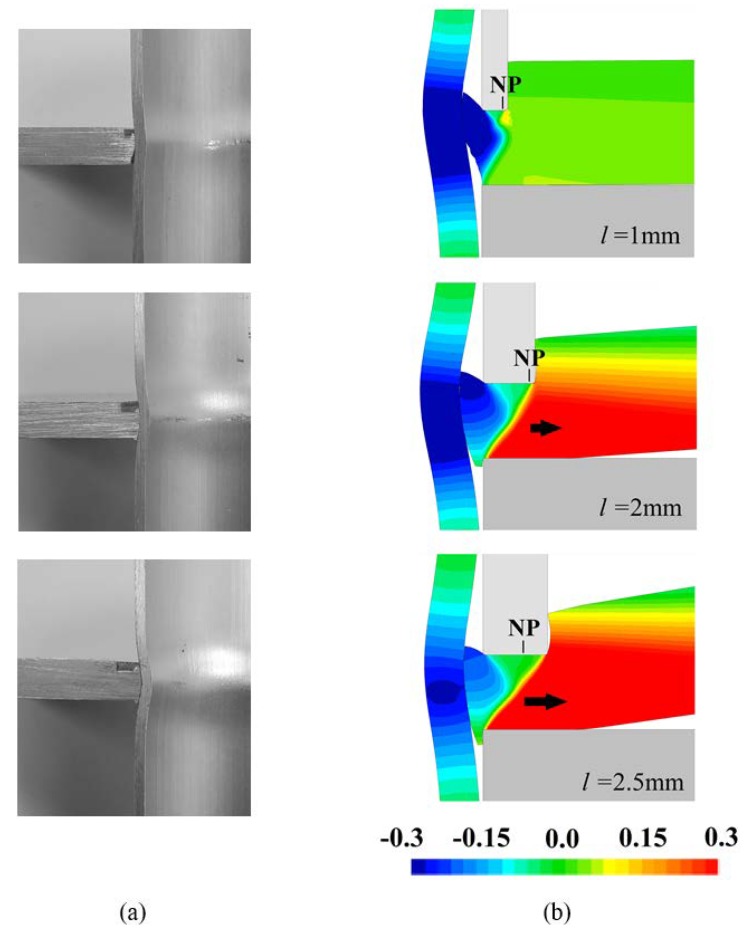
Influence of the cross-section recess length l of the punch on the form-fit joints for a squeezing depth d=2 mm. (**a**) Experimental cross-sections of the form-fit joints; (**b**) Finite element predicted cross-sections of the form-fit joints with the distribution of normalized radial velocity $v_{r}/v_{p}$.

This last conclusion is confirmed by the computed evolution of the normalized radial velocity $v_{r}/v_{p}$, where $v_{p}$ is the vertical punch velocity, shown in Figure 7b. As seen in case of d=2 mm, when the cross-section recess length of the punch increases from l=2 mm to l=2.5 mm, there is a shift of the neutral point (*NP*), corresponding to the transition between inward and outward material flow towards the left corner of the punch, meaning that more squeezed sheet material will start flowing outwards (refer to the black arrows included in Figure 7b).

Another consequence of the squeezed sheet material starting to flow outwards is the occurrence of bending. Bending gives rise to form-fit joints with a lack of perpendicularity between the sheet and the tube, as shown in the bottom experimental and numerically predicted cross-sections that are included in Figure 7a.

## 4. Discussion

### 4.1. Pull-Out Destructive Forces

The results obtained in Section 3 showed that both the cross-section recess length l and the squeezing depth d of the punch play a key role in the geometry of the inner tube bead of the form-fit joints. 

One question that naturally arises from Figure 6 is whether the correlation between l and d that maximizes the inner tube bead geometry of the form-fit joints is capable of ensuring the maximum pull-out forces that the joints can safely withstand before failing.

To answer this question, the authors took the form-fit joint with l=2 mm and d=2 mm lying close to the correlation line that maximizes the inner tube bead geometry and compared its pull-out destructive force with those obtained for other joints that were obtained by varying d along the dotted vertical line of Figure 6.

**Figure 8:**
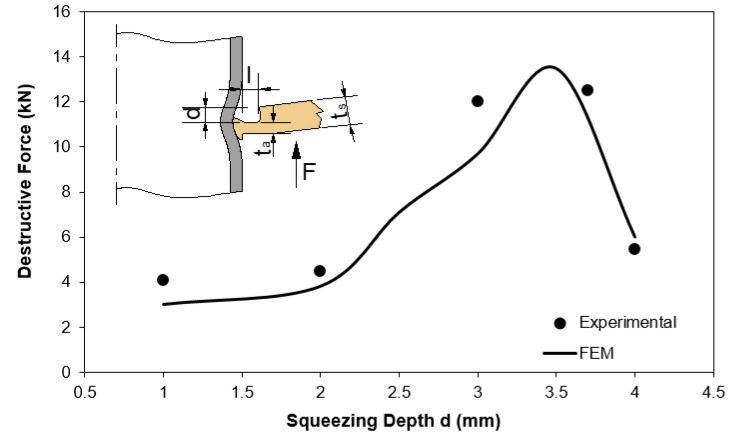
Influence of the squeezing depth d on the pull-out destructive force F of sheet–tube connections obtained with a punch having a cross-section recess length l=2 mm.

The results from experimental tests and numerical simulations are shown in Figure 8 and allow to conclude that the maximum pull-out destructive force F is not obtained for the process operating conditions that maximize the inner tube bead geometry of the form-fit joints.

Whilst at a first glance, this result may seem easy to explain because large values of d should give rise to larger inner tube beads of the form-fit joints, there is an additional phenomenon that also needs to be considered. Otherwise, it is not possible to fully understand the experimental and numerical results included in Figure 8, because the increase of the pull-out force F with d is only monotonic up to a peak, after which the force drops sharply.

The additional phenomenon that needs to be considered for understanding the evolution of the pull-out force F with d is the failure mechanism. In particular, the change in mechanism when the active sheet thickness $t_{a}$ left below the cross-section recess length of the punch (refer to the inset included in Figure 8) becomes very small.

**Figure 9:**
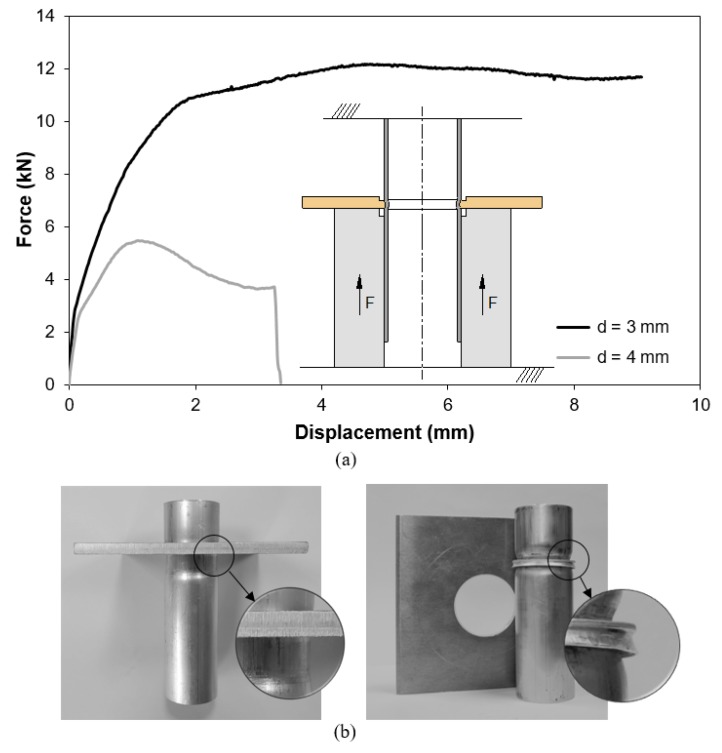
Pull-out tests. (**a**) Experimental evolution of the force with displacement for destructive pull-out tests performed in two different form-fit joints that were produced with a punch having a cross-section recess length l=2 mm. (**b**) Photographs of the two different tests specimens after failure by extrusion (left) and shearing (right).

Taking, for example, the pull-out force vs. displacement evolutions for the form-fit joints with d=3 mm and d=4 mm retrieved from Figure 8, it is easy to observe two totally different separation mechanisms (Figure 9a). One mechanism, leading to higher forces, is similar to tube extrusion while the other mechanism, leading to lower forces and a sharp drop at the end, is related to shearing.

In case of the first mechanism, observed in the pull-out test of the form-fit joint with d=3 mm, the sheet acts as a floating die and the tube is forced to plastically deform in order to reduce its inner radius from $r_{0}$ to $r_{b}$. The maximum pull-out force F attained in the destructive test is approximately equal to 12 kN.

The second mechanism, observed in the pull-out test of the form-fit joint with d=4 mm, is typical of shearing along the small active sheet thickness $t_{a}$ that was left below the cross-section recess length of the punch. The maximum pull-out force F associated to this mechanism is smaller than that of tube extrusion.

The photographs included in Figure 9b illustrate the differences between the two above-mentioned failure mechanisms. The conclusion to be taken from these tests is that the design of a joint to be produced by a punch having a cross-section length l=2 mm must consider a squeezing depth d=3−3.5 mm to ensure high pull-out destructive forces. 

In connection to this, it is worth mentioning that the concept of maximum pull-out force utilized by the authors refers to the force that prevents collapse by avoiding detachment of the two components and not to the force that is needed to produce the first relative movement between the sheet and the tube.

### 4.2. Sheet Thickness

**Figure 10:**
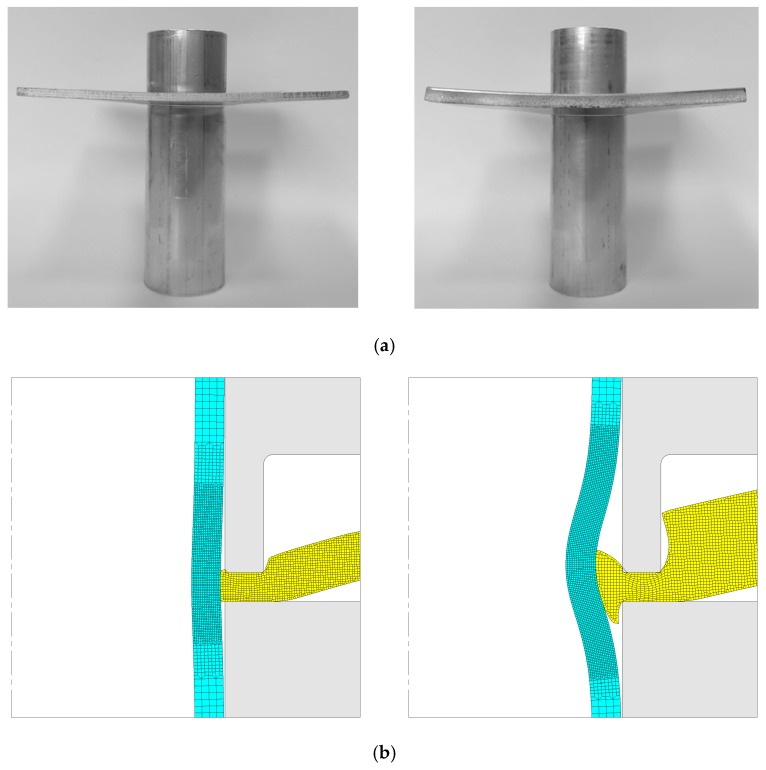
Mechanical joining of sheets to tubes by annular sheet squeezing using a punch with a cross section recess length l=2 mm and two different thicknesses $t_{s}=2.5$ mm and $t_{s}=5$ mm. (**a**) Photographs of sheets and tubes after being joined. (**b**) Finite element predicted cross section at the end of stroke for the two test cases shown in (**a**) using a squeezing depth d=1 mm (left) and d=3.5 mm (right).

The other question one may have about the mechanical joining of sheets to tubes by annular sheet squeezing is whether the process works for smaller sheet thicknesses than that utilized in the previous sections. To answer this question authors decided to produce sheet–tube joints using the same tube geometry but reducing the sheet thickness from $t_{s}=5$ mm to $t_{s}=2.5$ mm. The photograph included in Figure 10a shows that the process is still feasible for sheets with smaller thicknesses.

In view of the above, it is interesting for the readers to know which values of the cross-section recess length l and squeezing depth d of the punch were utilized to produce the form-fit joint with a sheet thickness $t_{s}=2.5$ mm. For this purpose, it is important to observe in Figure 8 that the maximum pull-out force of a form-fit joint produced with a punch having a cross-section recess length l=2 mm is obtained for a squeezing depth d=3.5 mm. Larger values of d, leading to active sheet thicknesses $t_{a}<1.5$ mm, provide smaller pull-out forces because the failure mechanism changes from extrusion to shearing. 

So, in order to use the same punch (l=2 mm) for comparison purposes, and prevent failure by shearing in case $t_{a}<1.5$ mm, it was decided to use a squeezing depth d=1 mm for obtaining the form-fit joint with a sheet thickness $t_{s}=2.5$ mm.

Figure 10b shows a detail of the computed finite element cross sections for the form-fit joints obtained for the two different sheet thicknesses. As seen, the magnitude of bending is significant in both cases, namely in the sheet with smaller thickness due to its lower stiffness. However, the phenomenon can be easily avoided by diminishing the gap between the cross-section recess length and the remaining flat surface of the punch so that the total amount of bending is limited.

## 5. Conclusions

Deformation-assisted joining of sheets to tubes by annular sheet squeezing is based on mechanical joining by means of form-fit joints. The amount of sheet material to be squeezed and the final shape and volume of the inner tube beads of the form-fit joints is controlled by the cross-section recess length l of the punch, the squeezing depth d and by the geometry of the punch and sheet, namely the sheet thickness $t_{s}$.

By varying these parameters, it is possible to change the plastic flow of the squeezed sheet material from a predominantly inward into a combination of inward and outward. In particular, it is possible to define a combination of parameters capable of ensuring maximum shapes and volumes of the form-fit joints for given values of d or l. However, the maximum shapes and volumes of the form-fit joints do not necessarily provide the maximum pull-out destructive forces because there is a minimum active sheet thickness $t_{a}$ below which the failure mechanism changes from extrusion to shearing and the forces drop significantly.

In connection to this, it is also shown that the new mechanical joining process is not limited to thick sheets because what needs to be fulfilled is a combination of parameters capable of ensuring a sound mechanical joint for values of the active sheet thickness $t_{a}$ above the critical threshold of failure by shearing.

## Figures and Tables

**Table 1:** The experiments on the mechanical joining of sheets to tubes by annular sheet squeezing. Notation is in accordance with Figure 2.

*r*_0_ (mm)	*t*_0_ (mm)	*t_s_* (mm)	*l* (mm)	*d* (mm)
14.5	1.5	2.5, 5	0.5–2.5	1.0–4.0
